# Underage drinking on saturday nights, sociodemographic and environmental risk factors: a cross-sectional study

**DOI:** 10.1186/1747-597X-6-15

**Published:** 2011-07-05

**Authors:** Luigi Gallimberti, Sonia Chindamo, Alessandra Buja, Giovanni Forza, Federica Tognazzo, Laura Galasso, Angela Vinelli, Vincenzo Baldo

**Affiliations:** 1 Azienda Ospedaliera di Padova, Toxicology Unit, Italy; 2 Department of Environmental Medicine and Public Health; Institute of Hygiene, University of Padova, Italy

## Abstract

**Background:**

Excessive alcohol consumption in underage people is a rising phenomenon. A major proportion of the disease burden and deaths of young people in developed nations is attributable to alcohol abuse. The aim of this study was to investigate social, demographic and environmental factors that may raise the risk of Saturday night drinking and binge drinking among Italian school students.

**Methods:**

The study was conducted on a sample of 845 Italian underage school students, by means of an anonymous, self-test questionnaire. Multivariate logistic regression was applied to identify independent risk factors for alcohol drinking and binge drinking. Ordered logistic regression was used to identify independent risk factors for harmful drinking patterns.

**Results:**

The independent variables that confer a higher risk of drinking in underage students are older age classes, male sex, returning home after midnight, belonging to a group with little respect for the rules, or to a group where young people are not seen as leaders. The higher the perception of alcohol consumption by the group, the higher the risk. Spending time in bars or discos coincides with a two-fold or four-fold increase, respectively, in the risk of alcohol consumption.

**Conclusion:**

Our findings show that certain environmental and social risk factors are associated with underage drinking. The most important role for preventing young people's exposure to these factors lies with the family, because only parents can exert the necessary control and provide a barrier against potentially harmful situations.

## Introduction

Despite clear evidence of the major contribution of alcohol to the global burden of disease and the related substantial economic costs, the attention paid to controlling alcohol consumption is still inadequate in most countries. The increasing industrial production and marketing of alcohol is driving a greater use of alcohol by young people too, and there are signs of rising levels of underage drinking [1]. A large proportion of the burden of disease and death among young people in developed nations is attributable to alcohol abuse: more than one in four deaths among males aged 15-29 can be attributed to alcohol, while the figure is 10% among females in the same age group [2]. Underage drinking, and binge drinking in particular, is associated with three of the main causes of death among young people: unintentional injury, homicide [3] and suicide [4] mainly in female [5]. Teenage drinking is also associated with higher risks of sexual assault and unprotected sex [2]. Alcohol consumption in adolescence may also cause permanent brain damage leading to problems with memory, learning capacity and verbal skills, as well as alcohol dependence and depression [6]. In defining social policies, decision-makers in various European countries and the EU as a whole have focused on teenage drinking, and much attention has been paid to risky drinking habits among young people - such as weekend binge drinking, which has been on the increase among young people across much of Europe in the last 10 years [7]. Significant research has been conducted on teenage drinking, addresses the related risk and protective factors. Great efforts have also been made to raise the awareness of families, schools and peers with a view to containing underage alcohol consumption. In particular, a study conducted in a representative sample of more than 14,000 school pupils (aged 7-11) in the UK revealed that poor parental supervision and discipline are associated with a higher probability of alcohol abuse [8]. Adolescents who do not fare well academically and who have a negative attitude to school are also at higher risk of drinking [9]. Many studies have highlighted the important role of peers in influencing health risk behavior [10].

Most of such studies concern young people in Northern Europe, while few studies have addressed drinking and the related risk factors in the underage population of the Southern European countries. Young people in the Mediterranean countries have traditionally been more likely to drink alcoholic beverages, and to drink them more often, than their counterparts in more northerly regions. At the same time, they were less likely to engage in excessive and extreme drinking patterns, get drunk, or exhibit other problem drinking behavior [2]. The times are changing, however, and young people in the Mediterranean countries now engage in binge drinking too [11]. The literature tends to generically consider drinking patterns without specifically assessing alcohol consumption on a particular week-end night, although the practice of drinking on a week-end night is associated with the most harmful effects of alcohol consumption. There is also a paucity of literature investigating the combined effects of socio-demographic and environmental risk factors relating to underage drinking. The aim of our study was to jointly investigate the social, demographic and environmental factors that might raise the risk of drinking among underage Italian school students.

Our findings may help policy-makers and planners in preventive public health to develop strategies for preventing alcohol use among young people, also because effective programs to prevent alcohol use among young adolescents can only stem from an understanding of the factors associated with its use in each particular population.

## Methods

### Participants

This survey was conducted in November 2008, in a province of the Veneto region in north-eastern Italy with a population of 442,023, including about 8,602 school students attending the 8^th ^grade, and about 14,400 attending the 9^th ^and 12^th ^grades. Italian law forbids the sale of alcoholic beverages to people under 16 years old.

The survey involved a sample of 845 underage students drawn from 15 schools in the province of Padua, who voluntarily took part in a school-based scheme for the prevention of underage alcohol consumption. Students at the schools participating in the scheme completed an anonymous baseline questionnaire administered by the team handling the prevention scheme. Informed verbal consent to the students' participation was required first from the school director, then from all parents and from each student enrolled. None of the parents or students refused. Non-respondents included students who were absent on the day of the survey for various reasons (illness, skipping lessons, job placements, etc.). Questions were asked in 13 classes of 8^th^-grade students (*n *= 239) at four lower secondary schools, in 21 classes of 9^th^-grade students (*n *= 437) at four upper secondary schools, and in 10 classes of 12^th ^-grade students (*n *= 163) at seven upper secondary schools. Three questionnaires were rejected because they failed to provide data on alcohol consumption. Six questionnaires did not indicate the students' ages and 35 did not state their gender.

### Materials

A new anonymous questionnaire was used, consisting of 43 multiple-choice questions consisting of words or numbers, with graphical illustrations to make them more appealing to this particular age group (52% of the students interviewed were very satisfied with the quality of questionnaire36% were moderately satisfied and 12% were dissatisfied). Briefly, respondents were asked for their basic demographic data (age and sex), social data (characteristics of their peer group and perception of alcohol use) and environmental information (people, places, and activities during the previous Saturday night, weekly pocket money, what time they returned home). The types and quantities of alcohol and non-alcoholic beverages consumed during the previous Saturday night were also assessed.

Information on alcohol use was collected by asking the following questions: How many glasses of wine did you drink last Saturday night?; How many bottles of beer did you drink last Saturday night?; and How many alcoholic cocktails did you have last Saturday night? The answers ranged from 0 to ≥5.

The following questions were asked to assess independent variables.

*How many people did you spent last Saturday night with ?*: "I did not spend it with a group", "with 2-4 friends", "with 5-9 friends", "with 10-20 friends", "in a group of more than 20 friends";

*What do you think about the group? Is it a group that you really want to belong to?*: "It is an important group that I want to belong to", "It is a group where I can fit in", "It is a group I tolerate", "It isn't a group that I want to spend time with";

*What is your group like?: *"We are very close", "quite close", "democratic", "in a subgroup", "Everyone is mischievous";

*What is your role within the group?*: "They do nothing without me", "I am important in my group", "I take part in the group", " I adapt to my group", "Nothing would change without me".

As for *the group's adherence to the rules*, the choice was between "sticking faithfully to the rules", "sticking partially to the rules", "being indifferent to the rules", "going partially against the rules", or "going entirely against the rules".

Students were also asked about the environment where they had spent the previous Saturday night: *Where did you spend last Saturday night? Who did you spend last Saturday night with? What did you do last Saturday night? *Respondents were asked to choose between various pre-codified alternatives also by means of graphical illustrations. The age variable was categorized in three groups: 12-13, 14-15, and 16-17 year-olds. Weekly pocket money was categorized in multiples of 10 Euro. Two more questions asked if they had drunk any alcohol on Saturday night and if they wanted to stop drinking alcohol on Saturday nights with a view to identifying their attitude to alcohol consumption on the assumption that any amount of alcohol in the underage population should be avoided. Finally, the students' perception of alcohol consumption by parents, peers, and their group was investigated by means of a visual scale from 0 to 10.

### Statistical methods

A bivariate analysis was initially carried out to illustrate the relationship between demographic, environmental, and behavioral variables and any alcohol drinking and binge drinking on the previous Saturday night. Statistical comparisons between two groups were drawn using Student's t-test for continuous variables and Wilcoxon-Mann-Whitney test when variable distributions did not meet assumptions for parametric testing. Statistical comparisons between more than two groups were drawn using Kruskal-Wallis Test when variable distributions did not meet assumptions for parametric testing. The Chi-square test has been applied for categorical variables and the Fisher's Exact Test only for categorical variables with expected frequencies less than 5. Relationships found significant at bivariate analyses were subsequently reassessed using a backward stepwise logistic regression model with an entry probability of 0.20 and an exit probability of 0.10. An ordered logistic regression was applied for ordered categorical outcomes such as drinking attitudes, considering different drink attitudes as ordinal modalities of a risk behavior assessment variable: Code 1 meant a student who never drank, code 2 an ex-drinker, code 3 a drinker who wanted to stop drinking, and code 3 a drinker who did not want to stop drinking. Statistical analyses were performed using the SPSS 16 and a p value of < 0.05 was considered significant.

## Results

### Distribution of alcoholic beverage consumers by gender and underage age group

We enrolled 845 students, 519 (61.64%) males and 288 (34.20%) females: 35 (4.16%) did not indicate their gender. The mean age of the sample was 14.46 (± 1.43) for the boys and 14.48 (± 1.59) for the girls. Among our 12-13 year-old students, 27.07% of the boys and 23.71% of girls had been drinking on the previous Saturday night: the figures rose to 35.57% of the boys and 32.71% of the girls aged 14-15 years, and to 72.66% of the boys and 46.46% of the girls 16-17 years old (Table 1). Men of almost any age group and type are more likely to consume alcoholic beverages than women and, in both sexes, older age coincides with a higher probability of becoming consumers.

**Table 1:** Relative frequency of consumers by sex and age group and different beverages

	12-13 years (n = 230; 113 M - 97 F)	14-15 years (n = 360; 253 M - 107 F)	16-17 years (n = 212; 128 M - 84 F)	*p*
**Beer**				
Males	18.05% (24/133)	25.30% (64/253)	46.88% (60/128)	<0.001
Females	17.53% (17/97)	11.21% (12/107)	21.43% (18/84)	0.15
**Wine**				
Males	11.28% (15/133)	12.25% (31/253)	20.31% (26/128)	0.06
Females	7.22% (7/97)	5.61% (6/107)	8.33% (7/84)	0.75
**Alcoholic cocktails**				
Males	19.55% (26/133)	20.95% (53/253)	46.09% (59/128)	<0.001
Females	16.49% (16/97)	24.30% (26/107)	33.33% (28/84)	0.03
**All alcoholic beverages**				
Males	27.07% (36/133)	35.57% (90/253)	72.66% (93/128)	<0.001
Females	23.71% (23/97)	32.71% (35/107)	46.46% (39/84)	0.005
**Binge drinking > 5 u.a**.				
Males	7.52 (10/133)	5.53 (14/253)	14.84 (19/128)	0.008
Females	3.09 (3/97)	0.00 (0/107)	4.76 (4/84)	0.09*

*p *for Chi-squared test _(2 df), _except for * where p for Fisher's exact test.

As for the students' drinking attitude, 39.01% were drinkers who did not want to stop; only 1.09% were drinkers who wanted to stop; 36.96% were ex-drinkers and 22.95% had never been drinkers.

### Perception of alcohol consumption in the student's social setting and underage misuse

Table 2 shows an evident association between alcohol consumption, binge drinking, drinking attitude and the perception of alcohol consumption by people in the student's social setting (parents, their group, and their peers generally).

**Table 2:** Drinking, binge drinking, and attitudes to drinking by perception of other people's alcohol consumption and dangers of drinking and driving

Drinking	Perception of alcohol consumption by				
	no drinking	yes drinking	p*		
**Parents**	2.5 (± 1.9)	3.2 (± 2.1)	<0.001		
**Peers**	4.5 (± 2.8)	6.3(± 2.5)	<0.001		
**Own group**	2.1 (± 2.5)	5.2(± 2.8)	<0.001		
**Dangers of drinking and driving**	9.4 (± 1.5)	8.9 (± 2.1)	0.004		
**Binge drinking**					
	no binge drinking	yes binge drinking	p*		
**Parents**	2.7 (± 2.0)	3.5 (± 2.7)	*0.06*		
**Peers**	5.1 (± 2.8)	7.1 (± 2.8)	<0.001		
**Own group**	3.1 (± 2.9)	6.5 (± 3.1)	<0.001		
**Dangers of drinking and driving**	9.3 (± 1.5)	7.9 (± 2.1)	0.009		
**Attitude to drinking**	drinkers who do not want to stop	drinkers who want to stop	ex-drinkers	non-drinkers	*p^§^*
**Parents**	3.5 (± 2.1)	2.4 (± 2.6)	2.8 (± 1.9)	1.9 (± 1.6)	<0.001
**Peers**	6.6 (± 2.3)	7.4 (± 2.6)	4.8 (± 2.7)	3.6 (± 2.8)	<0.001
**Own group**	5.5 (± 2.6)	5.3 (± 3.3)	2.4 (± 2.4)	1.1 (± 1.8)	<0.001
**Dangers of drinking and driving**	8.9(± 2.1)	9.2 (± 1.6)	9.4 (± 1.4)	9.5 (± 1.6)	<0.001

* p for Mann-Whitney U test_(1 df)_

^§ ^p for Kruskal-Wallis Test _(3 df)_

### Demographic, environmental and social factors associated with alcohol misuse in people underage

Bivariate analysis between Saturday night drinking and environmental and social factors showed that underage drinking on Saturday nights is associated with male sex, older age, more pocket money, and going home after midnight. Being alone reduced the probability of drinking alcoholic beverages, whereas spending time in a group or with a girl-or boy-friend on Saturday nights coincided with a higher likelihood of drinking. Staying at home also reduced the probability of alcohol consumption, whereas adolescents spending time in bars or discos, or wandering around, were more likely to drink alcoholic beverages. The activities associated with a lower likelihood of alcohol consumption were watching a film, playing on the computer, reading a book, while the activity associated with a higher probability of drinking was flirting. The type of company associated with higher alcohol consumption rates on Saturday nights included large groups, groups scarcely inclined to obey the rules, and groups in which young people were not perceived as leaders. On bivariate analysis the same environmental and social risk factors were associated with Saturday night underage binge drinking, except for students spending time with their group and wandering, while a new risk factor found associated with binge drinking, i.e. dancing. Table 3 shows the results of multivariate logistic regression. The independent variables conferring a higher risk of drinking by underage students are: older age, male sex, returning home after midnight, being part of a group scarcely inclined to obey the rules, or in a group where the young are not perceived as leaders. The risk is higher, the stronger the perception of alcohol consumption by the group. Spending time alone reduces the risk, while spending time in bars or discos carries a respectively two-fold and four-fold risk of drinking alcohol. The activity associated with the lowest probability of drinking is watching a film. The results of the multivariate logistic regression showed that the independent variables conferring a higher risk of binge drinking in underage adolescents are the same as for drinking generally, i.e. male sex, returning home after midnight, and being part of a group scarcely inclined to obey the rules. Here again, the risk is higher, the stronger the perception of alcohol consumption by the group. In addition, a higher risk of binge drinking coincided with a lower perception of the dangers of driving under the influence of alcohol. Spending time in discos carried a four-fold risk of binge drinking, and the same was true of flirting. The results of the ordered logistic regression showed that the independent variables conferring a higher risk of harmful drinking patterns are: older age, belonging to a group scarcely inclined to obey the rules, and not being a group leader: the risk is higher, the stronger the perception of alcohol use by the group and by the parents. Spending time in bars or in no particular place (just wandering around), and returning home after midnight raised the risk of adopting harmful drinking patterns. The factors associated with a lower risk were spending Saturday night alone, or watching a film or using the computer.

**Table 3:** Logistic regression models

**Logistic regression model 1**, **dependent variable: drinking on Saturday nights**					
		**OR**	**LL 95%CI**	**UL 95%CI**	p-value*

Female	D	0.653	0.438	0.972	*0.036*
Age group	D	1.332	0.999	1.776	*0.051*
Belonging to a group that breaks the rules	S	1.832	1.104	3.039	*0.019*
Not being the group leader	S	0.743	0.599	0.921	*0.007*
Perception of drinking by own group	S	1.375	1.273	1.484	*0.000*
Staying alone	E-pe	0.305	0.145	0.642	*0.002*
Going to bars	E-pl	2.700	1.302	5.597	*0.008*
Going to discos	E-pl	4.021	1.504	10.751	*0.006*
Watching a film	E-a	0.581	0.356	0.949	*0.030*
Returning home after midnight	E-a	1.878	1.132	3.114	*0.015*

**Logistic regression model 2**, **dependent variable: binge drinking on Saturday nights **(drink more than five alcoholic units)					

		**OR**	**LL 95%CI**	**UL 95%CI**	p-value*
Female	D	0.23	0.08	0.63	*0.004*
Belonging to a group that breaks the rules	S	2.77	1.30	5.89	*0.008*
Perception of drinking by own group	S	1.25	1.10	1.43	*0.001*
Perception of dangers of drinking and driving	S	0.84	0.74	0.96	*0.008*
Going to discos	E-pl	5.04	2.03	12.52	*0.000*
Flirting	E-a	4.28	1.92	9.53	*0.000*
Returning home after midnight	E-a	2.20	1.04	4.65	*0.039*

**Ordered logistic regression model 3, dependent variable: Attitudes to drinking **(non-drinker = 1, ex-drinker = 2, drinker who wants to stop = 3, drinker who does not want to stop = 4)					

		**OR**	**LL 95%CI**	**UL 95%CI**	p-value*
Age group	D	2.31	1.78	3.01	*0.000*
Not being the group leader	S	0.84	0.69	1.01	*0.080*
Belonging to a group that breaks the rules	S	1.98	1.25	3.16	*0.004*
Perception of drinking by own group	S	1.40	1.28	1.52	*0.000*
Perception of drinking by parents	S	1.21	1.11	1.32	*0.000*
Staying alone	E-pe	0.53	0.30	0.94	*0.024*
Being with own group	E-pe	0.60	0.41	0.90	*0.013*
Going to bars	E-pl	2.14	1.06	4.31	*0.033*
Wandering around	E-pl	1.52	1.05	2.20	*0.027*
Watching a film	E-a	0.68	0.44	1.07	*0.098*
Playing with the computer	E-a	0.40	0.23	0.69	*0.001*
Returning home after midnight	E-a	2.51	1.47	4.29	*0.001*

Abbreviation:

OR = adjusted odds ratio

LL 95%CI = lower limit 95% confidence interval

UL 95%CI = upper limit 95% confidence interval

D = demographic variables

S = social variables

E = environmental conditions (E-pe = people, E-pl = places and E-a = activities)

* p for Wald test_(1 df)_

## Discussion

This work first outlines the risk factors for drinking and binge drinking on Saturday nights among underage Italian adolescents. Our findings show that the proportion of drinkers on Saturday nights rises rapidly with age during adolescence, reaching 70% of 16-17 year-old males. There is also a significant alcohol consumption among youngsters under 16 (35.57% of the boys and 32.71% of the girls in age class 14-15), at an age when their consumption should be nil. These data indicate a higher prevalence of drinkers than the Italian national figures but these data agree with Regional Veneto prevalence esteems reported by National Centre for Epidemiology, Surveillance and Health Promotion of National Institute of Health [12], and they are consistent with findings in other countries. Another study on alcohol consumption by the young, the Australian School Students' Alcohol and Drug Survey, indicated that 34% of adolescents (14-17) had consumed alcohol in the previous week [13]. In the USA, 34.2% of boys and 24.2% of girls under the age of 13 reported drinking alcohol [14]. In 2009, the proportion of 8^th^, 10^th^, and 12^th ^graders who admitted to drinking alcoholic beverages in the month preceding a survey were 15%, 30%, and 44%, respectively [15]. These figures are alarming because such early alcohol use may have long-lasting consequences and early alcohol or other drug use is one of the most significant predictors of future alcohol dependence [16]. The brain undergoes major remodeling in adolescence, and alcohol abuse at this age may be associated with a reduction in the size of the hippocampus [17].

Our findings show that almost 15% of boys and 5% of girls aged 16-17 had been binge drinking on the previous Saturday night (which is higher than the national figures, indicating that 10.6% of boys and 3.9% of girls [18] report binge drinking once a year). This is a crucial issue, because this type of alcohol consumption can determine both acute and long-term damage. The acute effects may include a lack of readiness to react to external stimuli, uncertainties in balance and movement, and a reduced ability to control one's feelings, symptoms that can naturally lead to road accidents and to aggressive behavior that can end in injuries and violent or unprotected sex. Binge drinking also causes long-term damage and in adolescents it is the strongest predictor of excessive alcohol use in adulthood [19], as well as determining mood disorders (particularly depression), sleep disorders and sexual disorders.

In all the age groups considered, alcohol was used more by boys than by girls. This is consistent with nearly all the literature [20], though the difference between the genders is lower in young age brackets and higher among 15-16 year-olds.

There was no apparent difference in alcohol consumption between native Italians and adolescents of foreign origin, though our sample only included 8.31% of foreigners (n = 70) from 25 different countries, more than half of them from Eastern Europe, while other countries with different religions were not significantly represented.

The independent factors associated with drinking included both social and environment aspects (people, places and activities) relating to the adolescents' Saturday night pastimes. Saturday night at the disco raises the risk of adolescents drinking twice as much as spending time in bars. Parents should be encouraged to check on their adolescents' whereabouts and activities, discouraging these places as much as possible. On the other hand, 61% of young Australians said they drank alcohol at home [13]. Other factors associated with alcohol consumption include how long adolescents are allowed to stay out on Saturday nights, and returning home after midnight doubled the risk of drinking. Teenage drinking can be discouraged by parents monitoring this factor. Shawn also demonstrated that perceived parental monitoring and discipline have unique mediating capabilities, net the effects of all parenting behavior [21], while a lack of parentally-imposed structure and discipline has been found associated with a greater vulnerability to substance use among youths [22].

Peer influence is the other main factor related to alcohol consumption in young people [23] and our data confirm as much, showing the influence of the adolescent's perception of alcohol intake by the group, of the type of group with little respect for the rules as independent risk factors of drinking, and the protective effect of spending the evening alone. Young people's drinking is often seen as an attempt to achieve peer acceptance, though our data seem to indicate that some drink to gain the status of group leader because probably they think that alcohol give them vitality to draw the group.

Most of the independent risk factors for underage binge drinking on Saturday nights are the same as for their alcohol consumption generally; they are factors that drive the young to experiment with alcohol and also with its abuse. An additional risk factor for binge drinking is a lower perception of the dangers of driving under the influence of alcohol, this is probably due to the young that binge probably are that more habitually use alcohol so they fill more ensure and they have a reduction of risk perception due to precedent successfully tentative of driving after drinking. They do not have wise that incident is a binary event and in each bet you game the life. A surprising finding was that flirting is also an independent risk factor, probably because these youngsters use alcohol in order to feel less inhibited. This is an important issue, given the potential consequences in terms of unprotected sex and aggressive sexual behavior.

The attempt to analyze attitudes to alcohol consumption was made to establish the distribution of our young sample according to their drinking habits and any intention to stop drinking. The figures showed that our students tended to take extreme attitudes, and the young who drank did not intend to stop doing so in the future. There was nonetheless a group of youngsters who had used alcohol and then stopped, but this was probably not a carefully-meditated choice, but prompted by unpleasant contingent events. The sociodemographic factors associated with harmful drinking patterns were the same as for drinking and binge drinking on Saturday nights, with the addition of the students' perception of their parents' alcohol consumption. Young people's drinking behavior often emulates that of their parents: the more the adults drink, the more their children do too [24]. Positive role modeling is possibly the most important tool parents can use to convey information to their children about alcohol and its use.

The main limitation of this study lies in that the school sample was not randomly selected but based on a voluntary involvement of schools in the city of Padua, although all school are quested to be enrolled. The schools taking part in this study were therefore those more interested in alcohol prevention schemes, and the students attending these schools may consequently be more concerned about alcohol-related problems that general student population. These students however are more suited to the public health prevention programs on alcohol consumption.

Another limit of our study could be the adoption a new questionnaire, which was designed not to investigate a single domain but to give a description of several independent variables possibly associated with alcohol consumption. The questionnaire has a user-friendly layout and its easy-to-understand graphical presentation could considerably facilitate its use in research and in clinical practice.

## Conclusion

Among the risk factors identified, the most powerful predictors of underage drinking are spending time in places like discos and bars, but our findings showed that there are other environmental and social risk factors associated with adolescent alcohol consumption. The policy-maker consequently needs to focus on the regulations for selling alcohol in such places to people underage, and on enforcing such rules with severe penalties for guilty vendors. Our data also show that the attitude to alcohol of their group of peers has a strong influence on an adolescent's drinking habits. Perkins conducted a review of conceptual and empirical studies on the role of social norms in college student alcohol use, and prevention strategies to counter misuse [25]. Social norms refer to our perceptions and beliefs about what is 'normal' behavior. People may think their peers are heavy drinkers, and drink more themselves as a consequence, and yet much of peer influence is the result of erroneous perceptions. Normative feedback relies on presenting information on these misconceptions regarding personal drinking profiles, risk factors, and normative comparisons. A recent Cochrane meta-analysis suggested that policy-makers and prevention personnel use a web/computer feedback, which appeared to change normative perceptions of drinking and was possibly more effective among 15-to 24-year-olds students who drink for social reasons [26], though no evidence of its effectiveness has been produced for younger people. But the main agents potentially able to prevent youngsters' exposure to these risk factors of underage alcohol use are their parents [27]. First of all, parents have to drink responsibly, which means not drinking too much or too often, not only because the impression that parents have a high alcohol consumption is a risk factor, but also because it is only the family that can exert the necessary control and prevent children from finding themselves in harmful situations. To give an example, only a parent can establish the rule that an adolescent must return home before midnight. Parents also need to cooperate with other parents to monitor where their children meet (and oblige them to avoid dangerous places), and what they are doing, and to help them to find ways to enjoy themselves without alcohol [28]. Therefore families can influence their children directly with rules and restrictions where necessary, but also indirectly by selecting the environments with which their children interact, and by interpreting and responding to changing circumstances.

## Competing interests

The authors declare that they have no competing interests.

## Authors' contributions

Guarantors for the integrity of the study: LGallimberti, GF, VB; Study conception and design: VB, AB; Intellectual content: AB, LGallimberti, SC, LGalasso, FT; Statistical analysis: AB, AV. Data interpretation: LGalli, SC, AB, LGalasso, AV, VB. Manuscript writing: LGallimberti, SC, AB, AV, VB. All authors read and approved the final manuscript.
